# The influence of satisfaction on behavioral intentions in Chinese college students’ martial arts tourism: the mediating role of sport values

**DOI:** 10.3389/fpsyg.2026.1836354

**Published:** 2026-05-28

**Authors:** Yunlong Pang, Mei Han, Chao Liu

**Affiliations:** College of Physical Education, Qingdao University, Qingdao, China

**Keywords:** behavioral intention, martial arts tourism, satisfaction, sport industry, sport values

## Abstract

**Objectives:**

This study takes Chinese traditional sports - martial arts as the entry point, aiming to explore the mechanism by which tourists’ satisfaction in martial arts tourism affects their behavioral intentions, and to deeply analyze the mediating role of sports values between the two.

**Methods:**

A sample of 314 college students from seven universities in five provinces across the country was selected for this study. Data was collected through questionnaire surveys and analyzed. Data was analyzed using exploratory factor analysis, correlation analysis, multiple regression, and mediation analysis.

**Results:**

The results show that the satisfaction of college students with martial arts tourism has a significant positive impact on behavioral intention and sports values, and sports values play a partial mediating role between the two. Among them, the economic, social and aesthetic dimensions serve as positive mediators. The aesthetic dimension has the most significant effect. The physical dimension has an inhibitory effect. The mediating role of the moral dimension is not significant.

**Conclusion:**

These findings extend understanding of the psychological mechanisms underlying martial arts tourism participation and highlight the importance of value-based experiences in shaping future behavioral intentions. The study also provides practical implications for designing martial arts tourism products that better engage young adults.

## 1 Introduction

Martial arts, as a traditional sport, carries profound cultural significance. Throughout China’s long history, it has played an indispensable role. The inheritance and development of martial arts have gradually become a uniquely distinctive cultural treasure within China’s outstanding traditional culture (Wang, 2025). Promoting martial arts culture essentially represents a multidimensional elevation and transmission of sports values. As a comprehensive belief system, sports values encompass people’s distinctive perspectives, convictions, and attitudes toward the worth of sports, playing a crucial guiding role in shaping individuals’ sports-related ideologies and behavioral patterns (Wang Y., 2024). In the current environment, martial arts culture faces dual crises—internal and external. Internally, identity conflicts and a narrow focus on combat techniques have led to a lack of cultural trust. Externally, it faces challenges from Western sports culture and global combat sports (Zhao, 2018). With the advent of the digital age, the inheritance and development of martial arts encounter new challenges alongside new opportunities (Xie and Luo, 2025). To give an example, during the era of technologies, video games may be used to demonstrate the spirit of classic martial arts and popularize the spirit of Chinese sports (Bai and Zhang, 2025). On the same note, an Internet Plus Education research perspective offers the possibility to explore the value of its application in college martial arts education (Duan, 2024). All these produce the following effect: The evolutionary course of martial arts is experiencing an unprecedented change. One of the approaches to the further development of martial arts should include the integration of martial arts with modern technology and marketing strategy to expand martial arts spread networks and reach the entire audience, and hence, enhance martial arts culture.

With the growing importance of quality of life and personal fulfillment among people, standard tourism paradigms are slowly changing into experiential and immersive ones. In light of the present-day unparalleled combination of cultural tourism and martial arts, martial arts tourism has been drawing considerable attention. In this study, martial arts tourism refers to a form of sport-cultural tourism in which participation in martial arts, learning, performance viewing, and cultural experience constitute the core or an important part of the trip. This definition is grounded in the understanding of sports tourism in Chinese scholarship, namely, that sports tourism is a type of tourism centered on sports resources, sports participation, and sports spectatorship, and it is also consistent with the policy direction emphasized in China of promoting the deep integration of martial arts and tourism and developing diversified martial arts products and services (Xie et al., 2021; General Administration of Sport of China et al., 2019). At present, representative forms of martial arts tourism in China include visits to the Shaolin Temple and watching various types of martial arts touring performances. Other notable thematic activities include the “Chinese Kung Fu Experience Tour” cultural event launched by the Henan Provincial Department of Tourism on 23 July 2019, and the World Wudang Tai Chi Congress held in Wudang Mountain in October 2023. As a new form of integration martial arts tourism is gaining popularity in all the sectors of society. It is against this background that the General Administration of Sport of China, together with 14 departments, such as the Ministry of Culture and Tourism, announced the publication of the plan of martial arts industry development (2019–2025). This document shows clearly that there should be a profound integration between martial art and tourism that should promote the generation of martial arts products in terms of tourism. With its unique appeal and multifaceted value, martial arts tourism has become a key pathway for expanding new tourism formats and promoting martial arts culture.

In the process of analyzing future market trends, the college student group has inevitably become a key point of focus. Based on the latest “2024 China College Student Consumption Behavior Survey Report” issued by iiMedia Research, a global third-party data mining and analysis organization specializing in the new economy industry, the number of higher education enrollments in China has reached 47.63 million students and is still on the rise. Among them, 41.1% of college students have an average monthly income between 1,501 and 2,000 yuan, with an estimated annual consumption scale of approximately 850 billion yuan in 2024, which shows strong consumption potential. In addition, with the development of society and the passage of time, the spiritual and cultural needs of modern college students are becoming increasingly diversified and individualized. Once their material needs are met, they are more interested in spiritual pursuits and have a strong interest in sports and fitness, leisure, and entertainment. A visualization analysis of literature on college students’ sports consumption from the CNKI database reveals that “college students,” as one of the core keywords, appears 329 times with a centrality of 0.7, highlighting this group’s significance in sports consumption (Liu, 2024). Simultaneously, within the tourism market, university students stand out among other consumer groups for their abundant travel time, high energy levels, strong adaptability, and immense market potential (Yu, 2022). Moreover, this demographic is youthful and dynamic, possesses strong cultural identification, high social needs, and considerable consumption capacity and influence. Their educational background and learning abilities equip them with more advanced sports values, making them more willing to deeply understand and experience martial arts culture. By participating in martial arts tourism, they cannot only effectively promote martial arts culture and drive tourism product innovation but also stimulate local economic development and enhance cultural exchange, playing a unique and positive role in advancing both martial arts and tourism. Therefore, this study focuses on Chinese college students as the target population. By observing and analyzing their behaviors, it aims to explore how satisfaction in martial arts tourism influences behavioral intentions through the mediating role of sport values.

Although previous studies have examined martial arts tourism, satisfaction in martial arts tourism, and behavioral intentions to some extent, the existing literature has mainly focused on macro-level issues such as industrial development, cultural communication, resource development, and integration pathways of martial arts tourism, while paying insufficient attention to the internal psychological mechanism through which martial arts tourism experiences further influence individual behavioral intentions. In particular, among Chinese college students, an important potential consumer group, empirical research on the relationships among satisfaction in martial arts tourism, sport values, and behavioral intentions remains limited, and the possible mediating role of sport values has not been sufficiently tested. Against the background of the continued deep integration of martial arts and tourism, as well as the growing demand among college students for cultural consumption and experiential tourism, exploring this issue is of strong practical urgency and theoretical significance. For these reasons, the purpose of this study is to take Chinese college students as the research population, examine the effect of satisfaction in martial arts tourism on behavioral intentions, and further test the mediating role of sport values, so as to reveal the internal mechanism through which martial arts tourism experiences influence behavioral intentions. The contributions of this study are reflected in three aspects. First, it extends the analytical framework of martial arts tourism research from the perspective of sport values. Second, it deepens the understanding of the internal mechanism through which satisfaction affects behavioral intentions. Third, it provides empirical evidence for the development and optimization of martial arts tourism products, communication pathways, and promotional strategies targeting college students.

## 2 Literature review and hypothesis development

### 2.1 Theoretical foundations

To better construct the overall logical framework of this study it is necessary to be explained how satisfaction in martial arts tourism is related to sport values and behavioral intentions in terms of theory.

According to the Expectation-Confirmation Theory (Oliver, 1980), individuals compare actual experience with prior expectations. When the experience surpasses the expectations, satisfaction is created, and this satisfaction in its turn affects the subsequent attitudes and behavioral preferences. Hence, satisfaction not only in the context of martial arts tourism is a direct assessment of the experience of tourism, but also a significant antecedent of behavioral intentions like returning and sharing the experience with the others. Meanwhile, Flow Theory (Csikszentmihalyi, 1975) is the viewpoint that individuals are more likely to develop positive emotional assessment and reinforce an intention to engage in an activity when they experience such a high degree of involvement, concentration and pleasure. Such positive experiences can be stimulated by martial arts tourism that is an activity involving physical activity, cultural experience and immersion into the situation, which support the role of satisfaction on behavioral intentions further.

Social Cognition Theory (Bandura, 1986) underscores the idea that by processing external experiences cognitively, individuals form judgments, beliefs, and behavioral tendencies. It suggests that the perceived experiences in tourism do not stay at the level of immediate sensation, but have the possibility of forming the value perception of sport among individuals. Theory of Consumption Values (Sheth et al., 1991) further argues that people take various dimensions into consideration in making behavioral decisions, including functional value, social value, emotional value and cognitive value. Regarding martial arts tourism, economic, social, aesthetic, physical and moral value perception that a tourist develops in the experience can be a major psychological foundation to their future intentions to behave in a certain manner.

**FIGURE 1:**
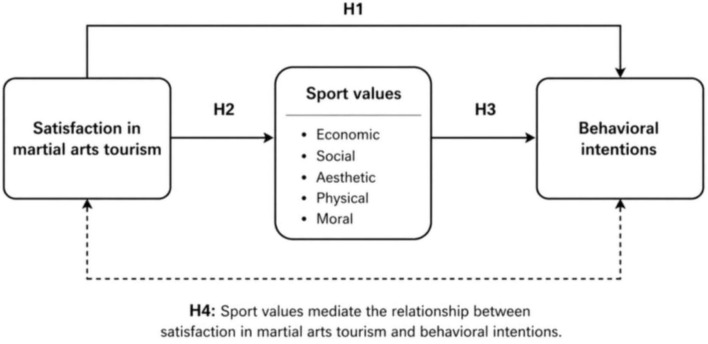
Conceptual framework of the study.

Based on the above theories, this study adopts an integrated multi-theoretical perspective, regarding satisfaction as the antecedent variable, sport values as the mediating variable, and behavioral intentions as the outcome variable, in order to explain the internal mechanism underlying martial arts tourism experiences. On this basis, the following four research hypotheses are proposed. The conceptual framework of the study is presented in Figure 1.

H1. Satisfaction in martial arts tourism significantly and positively influences tourism behavioral intentions.

Early studies have shown that overall satisfaction influences tourists’ behavioral intentions. Therefore, to systematically investigate behavioral intentions, research on tourists’ overall satisfaction needs to be further refined. The academic community has developed a relatively clear understanding of the formation mechanism of tourists’ behavioral intentions. For example, Allameh et al. (2015) revealed the following causal chain: destination image shapes perceived quality, perceived quality affects perceived value, perceived value then determines satisfaction, and satisfaction ultimately influences revisit intention. Within this framework, tourism satisfaction plays a crucial role in linking tourists’ initial experience perceptions with their subsequent behavioral tendencies. In the context of sports tourism, positive experiences often strengthen tourists’ emotional connection with the destination and increase their willingness to revisit and recommend it to others. As Feng and Tian (2024) pointed out, tourists’ satisfaction or dissatisfaction directly affects whether they are willing to return or recommend the destination. Positive emotions generated during the tourism experience can further promote identity, attachment, and favorable behavioral responses. In addition, Hermann et al. (2025)’s research also suggests that variables such as expectations, experience, quality perception, satisfaction, and willingness to return are closely related. When tourists perceive that their actual experience matches or exceeds their prior expectations, they are more likely to form positive evaluations of service quality, experience satisfaction, and revisit intention.

H2. Satisfaction in martial arts tourism significantly and positively influences sport values.

Zeng et al. (2017) studied Chinese tourists participating in sports activities on Jeju Island. By constructing a sports tourism service quality influence model, they found that the quality of hardware environments and experiential outcomes in sports tourism settings significantly and positively drive tourist satisfaction. This satisfaction further reinforces tourists’ cognitive dimensions of sports tourism’s “leisure value” and “experiential value.” Akhoondnejad (2018) empirically validated through structural equation modeling that the process of tourism satisfaction formation serves as a core vehicle for permeating sports values. When tourists derive satisfaction from event quality and emotional experiences, they actively reinforce positive perceptions of horse racing as a sporting activity, thereby solidifying related sports values. Based on this, this study further proposed the second hypothesis: In the specific context of martial arts tourism, the satisfaction of tourists may also influence tourists’ sport values. This hypothesis was developed based on the above discussion. That is to say, the satisfaction of tourists was not only considered to be one of the important motivations for the development of sports tourism but also, through the actual experiences of the tourists, may subtly influence individuals’ cognitive patterns and value judgments toward sports.

H3: Sport values significantly and positively influence tourism behavioral intentions.

According to Yu et al. (2017), values can be understood as a set of internalized belief systems about how an individual should live and what is worth pursuing. These beliefs not only provide basic standards for judging the importance of things and the usefulness of behavior, but also serve as an important reference for individual value judgment. In tourism research, values also play an important role because they are deeply rooted in an individual’s cognitive structure, influencing destination preferences and behavior during the tourism experience. Schoeman et al. (2016) showed that different tourist groups display different behavioral preferences according to their value orientations. For example, experienced divers tend to place greater emphasis on exploration, challenge, and the cognitive and professional dimensions of diving activities, whereas amateur participants are more likely to view the activity as entertainment and relaxation. These differences in value orientation further shape their participation choices and behavioral tendencies. Similarly, Han et al. (2023), in the context of a major college football event in the United States, found that tourists with a high social value orientation were more likely to regard the sports event as a way to connect with others who shared similar backgrounds or interests. In this case, the event functioned not only as a viewing experience, but also as a medium for gaining social belonging. Such value-based emotional identification and social attachment can strengthen participation motivation and behavioral intention. Therefore, based on the above studies, this paper proposes the following hypothesis:

H4: Sport values mediate the relationship between satisfaction in martial arts tourism and behavioral intentions.

Based on the above analysis, it can be seen that satisfaction in martial arts tourism not only directly influences individuals’ behavioral intentions, but also further affects their sport values, while sport values in turn exert a significant influence on behavioral intentions. That is, the satisfaction impact cannot only be an immediate positive analysis; it may also extend to and shape future behavioral decisions, by strengthening individual cognition, recognition and perception of martial arts tourism and sport. Despite the fact that previous studies have been able to individually analyze the relationship between satisfaction, value perception and behavioral intentions, few studies have incorporated the three variables into a unified framework and systematically test the internal process of the variables. To the Chinese college students, martial arts tourism is a comprehensive activity that involves physical activity, culture, as well as socialization. When they come to a highly satisfactory level when touring martial arts, they are more likely to develop positive sport value cognition which in the end increases their readiness to come back, give recommendations and proceed to participate. As such, sport values are likely to mediate the relationship between martial arts tourism satisfaction and behavioral intentions.

## 3 Research methods

### 3.1 Research participants

In this research, the questionnaire survey was conducted through a multi-stage purposive sampling method based on geographical distribution. To enhance its geographical coverage, first, the regions of the surveys were chosen based on various geographical locations in China. Second, survey locations were the target universities in these areas. Lastly, cooperating teachers at each university assisted in distributing the questionnaires to the appropriate college students that were engaged or had a related experience with the martial arts tourism activities. The geographical distribution became the primary sampling factor in this study, and other variables were not applied (such as major and gender) to define stratification further. It was primarily due to the fact that the respondents needed to fulfill the eligibility criterion of possessing relevant martial arts tourism experience and that the pool of qualified participants was relatively limited. In this process the study has tried to enhance the region coverage and increase the region representativeness.

To investigate how satisfaction in martial arts tourism influences behavioral intentions through sport values among college students during martial arts tourism, questionnaires were distributed to and collected from a total of 314 research subjects, with 314 valid responses obtained. Considering regional variations in martial arts tourism participation across China, universities in five provinces Beijing, Anhui, Shaanxi, Shandong, and Guangxi were selected. To achieve geographical uniformity among research subjects, Students from seven universities Qingdao University, China University of Political Science and Law, Capital University of Physical Education and Sports, Shaanxi University of Science and Technology, Guangxi Normal University, Anhui University of Finance and Economics, and Shandong Sport University were selected using multi-stage purposive sampling. Detailed participant information is presented in Table 1.

**TABLE 1:** Basic characteristics of the study population.

Variable	Category	Total	Percentage
Gender	Male	123	39.17%
	Female	191	60.83%
Grade level	1st grade	5	1.59%
	2nd grade	225	71.66%
	3rd grade	49	15.61%
	4th grade	33	10.51%
	4th grade and above	2	0.64%
Major field	Humanities and social sciences	86	27.39%
	Education	18	5.73%
	Engineering	86	27.39%
	Natural sciences	27	8.6%
	Medicine and pharmacy	37	11.78%
	Arts and physical education	33	10.51%
	Other	27	8.6%

### 3.2 Questionnaires

The questionnaire used in this study consisted of 43 items (the questionnaire includes eight questions on satisfaction in martial arts tourism, five questions on tourism behavioral intentions and 30 questions on sports values), all of which were adapted from established scales in previous studies rather than being self-developed. Specifically, the satisfaction in martial arts tourism scale was used in the studies of Nguyen Huu et al. (2024), Lee et al. (2007), Gallarza and Gil Saura (2006), Patterson and Spreng (1997), Mano and Oliver (1993), Yi (1990). The behavioral intentions scale mainly referred to the studies of Tian and Jebbouri (2025), Duman and Mattila (2005), Tsai (2005). The sport values scale mainly drew on the related studies of Zheng (2008), He and Ling (2024). Based on the original scales, several items were slightly revised to fit the context of martial arts tourism among Chinese college students, so as to enhance the contextual relevance and comprehensibility of the scale.

In this study, exploratory factor analysis was conducted to examine the construct validity of the scales measuring satisfaction in martial arts tourism, tourism behavioral intentions, and sport values. All items met the validity criteria and were retained. Satisfaction in martial arts tourism and tourism behavioral intentions each formed a single factor, while sport values were further classified into five factors: economic, social, aesthetic, physical, and moral values. Eigenvalues for the seven factors ranged from 2.609 to 5.559, with the variance explained by each factor ranging from 65.228% to 81.811%, indicating satisfactory construct validity. The Kaiser-Meyer-Olkin (KMO) measure of sampling adequacy was.946, and Bartlett’s test of sphericity was significant (χ^2^ = 9733.918, *p* < 0.001), confirming that the data were suitable for factor analysis. Reliability (Cronbach’s α) ranged from 0.819 to 0.937 across the seven factors, demonstrating high internal consistency. Detailed factor loadings and reliability coefficients are presented in Table 2.

**TABLE 2:** Validity and reliability.

Item	Satisfaction	Behavioral intentions	Economic	Social	Aesthetic	Physical	Moral	Reliability	Eigenvalue	Variance (%)	Cumulative (%)
S1	0.822	0.120	0.100	0.146	0.171	0.209	0.177	0.926	5.559	69.483	69.483
S2	0.830	0.177	0.133	0.082	0.159	0.248	0.118				
S3	0.779	0.112	0.087	0.145	0.262	0.227	0.149				
S4	0.782	0.147	0.126	0.157	0.217	0.267	0.114				
S5	0.772	0.086	0.141	0.230	0.212	0.249	0.111				
S7	0.570	0.314	0.157	0.246	0.274	0.185	0.083				
S8	0.725	0.258	0.092	0.038	0.159	0.128	0.138				
B2	0.321	0.741	0.185	0.142	0.328	0.074	0.154	0.897	3.598	71.959	71.959
B3	0.304	0.760	0.174	0.190	0.275	0.140	0.202				
B4	0.251	0.719	0.227	0.248	0.227	0.241	0.121				
B5	0.319	0.712	0.214	0.163	0.279	0.186	0.227				
V1	0.132	0.136	0.816	0.076	0.150	0.085	0.065	0.819	2.609	65.228	65.228
V2	0.080	0.132	0.822	0.075	0.074	0.110	0.052				
V4	0.192	0.063	0.642	0.071	0.348	0.101	0.137				
V5	0.078	0.315	0.561	0.325	0.198	0.198	0.020				
V7	0.169	0.218	0.175	0.728	0.147	0.323	0.179	0.925	3.272	81.811	81.811
V8	0.158	0.261	0.102	0.705	0.242	0.255	0.299				
V9	0.135	0.220	0.064	0.652	0.233	0.484	0.203				
V10	0.236	0.189	0.109	0.620	0.264	0.429	0.166				
V16	0.177	0.222	0.218	0.207	0.633	0.194	0.136	0.878	3.387	67.746	67.746
V17	0.192	0.143	0.164	0.200	0.574	0.466	−0.080				
V18	0.261	0.134	0.079	0.292	0.691	0.126	0.166				
V19	0.260	0.147	0.150	0.129	0.648	0.251	0.224				
V20	0.235	0.209	0.132	0.203	0.624	0.262	0.329				
V21	0.192	0.136	0.094	0.164	0.188	0.745	0.169	0.937	4.020	80.402	80.402
V22	0.206	0.102	0.100	0.169	0.181	0.818	0.071				
V23	0.214	0.123	0.121	0.187	0.109	0.831	0.105				
V24	0.194	0.072	0.115	0.145	0.097	0.838	0.186				
V25	0.209	0.082	0.116	0.152	0.117	0.839	0.149				
V27	0.190	0.227	0.203	0.151	0.143	0.378	0.686	0.903	3.115	77.865	77.865
V28	0.176	0.196	0.162	0.190	0.258	0.176	0.768				
V29	0.247	0.172	0.027	0.225	0.315	0.417	0.585				
V30	0.252	0.109	0.083	0.182	0.390	0.269	0.627				

Kaiser-Meyer-Olkin measure of sampling adequacy = 0.946, Bartlett’s test of sphericity = 9733.918, *p* = 0.000.

### 3.3 Data processing

Data analysis was performed using SPSS 25 and PROCESS 3.3, as follows:

(1) Frequency analysis was conducted to identify the demographic characteristics of the participants.

(2) Reliability analysis was performed to assess the internal consistency of the questionnaire.

(3) Exploratory factor analysis (EFA) was conducted to examine the factor structure of the scales.

(4) Pearson correlation analysis was used to examine the relationships among satisfaction in martial arts tourism, sport values, and behavioral intentions.

(5) Multiple regression analysis was performed to test the direct effects among the variables.

(6) Mediation analysis was conducted to examine the mediating role of sport values in the relationship between satisfaction in martial arts tourism and behavioral intentions.

## 4 Results

### 4.1 Correlation analysis

In this study, Pearson correlation analysis was conducted to examine the relationships among satisfaction in martial arts tourism, tourism behavioral intentions, and the five dimensions of sport values. According to Table 3, satisfaction in martial arts tourism and tourism behavioral intentions showed a strong positive correlation (*r* = 0.647, *p* < 0.001). Satisfaction in martial arts tourism was also significantly associated with all five dimensions of sport values, including economic (*r* = 0.427, *p* < 0.001), social (*r* = 0.555, *p* < 0.001), aesthetic (*r* = 0.642, *p* < 0.001), physical (*r* = 0.554, *p* < 0.001), and moral values (*r* = 0.579, *p* < 0.001). Similarly, tourism behavioral intentions exhibited significant correlations with economic (*r* = 0.533, *p* < 0.001), social (*r* = 0.610, *p* < 0.001), aesthetic (*r* = 0.673, *p* < 0.001), physical (*r* = 0.460, *p* < 0.001), and moral values (*r* = 0.602, *p* < 0.001). All dimensions of sport values were also positively interrelated with one another. These results indicate stable and meaningful associations among the core variables, as detailed in Table 3.

**TABLE 3:** Pearson correlation analysis.

Variable		Satisfaction	Behavioral intentions	Sport values				
				Economic	Social	Aesthetic	Physical	Moral
Satisfaction		1	–	–	–	–	–	–
Behavioral intentions		0.647***	1	–	–	–	–	–
Sport values	Economic	0.427***	0.533***	1	–	–	–	–
	Social	0.555***	0.610***	0.447***	1	–	–	–
	Aesthetic	0.642***	0.673***	0.528***	0.666***	1	–	–
	Physical	0.554***	0.460***	0.369***	0.668***	0.576***	1	–
	Moral	0.579***	0.602***	0.429***	0.653***	0.690***	0.604***	1

***p < 0.001.

### 4.2 Multiple regression analysis

The results of the multiple linear regression analysis in this study are presented in Table 4, the analysis showed that satisfaction in martial arts tourism had a significant predictive effect on sport values with a correlation coefficient of R = 0.698, explaining 48.8% of the variance. The regression model was statistically significant (F = 293.929, *p* < 0.001). The direct effect of Satisfaction in martial arts tourism on sport values was strong and significant, with (β = 0.698 and *t* = 17.144). Therefore, H2 proposed in this study are supported.

**TABLE 4:** Multiple regression analysis among satisfaction, sport values, and behavioral intentions.

Variable		R	R^2^	F	β	*t*
Sport values	Satisfaction	0.698	0.488	293.929***	–	14.726***
		–	–	–	0.698	17.144***
Behavioral Intentions	Satisfaction	0.768	0.590	221.417***	–	−5.613***
		–	–	–	0.339	6.643***
	Sport values	–	–	–	0.492	9.656***

***p < 0.001.

For Tourism behavioral intentions, the analysis revealed a significant overall association with its predictors, yielding R = 0.768 and explaining 59.0% of the variance. The regression equation was significant (F = 221.417, *p* < 0.001). The total effect path demonstrated a significant coefficient (*t* = −5.613). When satisfaction in martial arts tourism and sport values were entered simultaneously, both exerted significant positive effects on Tourism behavioral intentions, with Satisfaction in martial arts tourism (β = 0.339, *t* = 6.643) and sport values (β = 0.492, *t* = 9.656) showing substantial explanatory power. H1 and H3 proposed in this study were supported. The detailed results for each hypothesis are presented in Table 5.

**TABLE 5:** Hypothesis testing.

No.	Hypothesis	Test result
1	Satisfaction → behavioral intentions	Accepted
2	Sport Values → behavioral intentions	Accepted
3	Satisfaction → sport values	Accepted
4	Satisfaction → sport values → behavioral intentions	Accepted

### 4.3 Mediation analysis

To further explore on the relationship between sport values and martial arts tourism satisfaction, the mediation analysis was carried out on the five sub-dimensions of sport values to determine whether the five sport value dimensions were mediating in the relationship between satisfaction in martial arts tourism (X) and tourism behavioral intentions (Y). The overall impact of satisfaction in martial arts tourism on tourism behavioral intentions was found to be significant as indicated in Table 6 (Effect = 1.096, BootSE = 0.074, 95% BootCI = [0.952, 1.241]). The direct effect remained significant after adding the mediators (Effect = 0.509, BootSE = 0.086, 95% BootCI = [0.339, 0.679]) which implies partial mediation.

**TABLE 6:** Mediation analysis.

Path	Effect	BootSE	95% BootCI lower	95% BootCI upper	Standardized effect
Total effect (X→Y)	1.096	0.074	0.952	1.241	0.647
Direct effect (X→Y)	0.509	0.086	0.339	0.679	0.300
Indirect effect (X→M→Y)					
Economic	0.132	0.045	0.053	0.230	0.078
Social	0.194	0.073	0.057	0.344	0.114
Aesthetic	0.244	0.075	0.102	0.395	0.144
Physical	−0.114	0.057	−0.231	−0.008	−0.067
Moral	0.132	0.071	−0.006	0.272	0.078
Total indirect effect	0.587	0.080	0.437	0.749	0.346

Bootstrap sample size = 5,000. All continuous variables were mean-centered prior to analysis.

Considering indirect effects, this echoed through the three dimensions of sport values where economic, social and aesthetic values exhibited high positive mediating effects. Namely, the indirect effect on economic, social, and aesthetic values was 0.132 (BootCI [0.053, 0.230]), 0.194 (BootCI [0.057, 0.344]), and 0.244 (BootCI [0.102, 0.395]), respectively. The confidence intervals of these effects did not include zero, confirming statistical significance.

In contrast, the indirect effect of physical values was negative (Effect = −0.114) with a 95% BootCI of [−0.231, −0.008], indicating a suppressing effect. The indirect effect of moral values (Effect = 0.132) did not reach significance, as its confidence interval included zero (BootCI [−0.006, 0.272]).

The total indirect effect across all five mediators was significant (Effect = 0.587, BootSE = 0.080, 95% BootCI [0.437, 0.749]), Standardized effects ranged from −0.067 to 0.144 across individual mediators, with a total standardized indirect effect of 0.346. Representing a meaningful mediating role of sport values in the relationship between satisfaction in martial arts tourism and tourism behavioral intentions, therefore, H4 was supported.

## 5 Discussion

Based on the specific context of martial arts tourism, this study has proposed a model of “experience-value-satisfaction-behavioral intention,” the results indicate that the sports value plays a bridging role in the process from satisfaction to behavioral intention, i.e., the process from satisfaction to revisiting does not occur directly, but also requires the capability to activate value recognition, this finding is not only interesting in the elaboration of the theory of martial arts tourism, but also in the implementation and development of the same in the future. From this model, we can see that this reflects the overall psychological process: how does one start with external experience and gradually accumulate it in their mind and finally arrive at the point where they create their own identity and make the decision? That is the process from one step to another, and if we speak about this in the context of real people, this is how behavior is formed. Therefore, the present study extends existing research by highlighting the mediating role of sport values and by offering a clearer explanation of the psychological mechanism underlying behavioral intention in martial arts tourism.

### 5.1 The relationship between satisfaction in martial arts tourism and tourism behavioral intentions

#### 5.1.1 Satisfaction in martial arts tourism can significantly and positively enhance tourism behavioral intentions

This study confirms that satisfaction in martial arts tourism exerts a significant positive influence on tourism behavioral intention. According to Nguyen Huu et al.’s (2024) perspective, when tourists’ actual experiences meet or exceed their previous expectations, a sense of psychological satisfaction will arise, which in turn stimulates positive behavioral intentions for tourism. Nevertheless, a simple summary of this relationship as a simple satisfaction–intention link is most of the times misleading of the actual value and deeper inspirations of the experiential forms. Therefore, we refer to a more comprehensive concept, that is, as long as a person is highly engaged in an action, individuals may enter a state of enjoyment and immersion. The focus on, attending, and sensing martial arts activities bring satisfaction and pleasure to martial arts tourists. This increases their satisfaction with the tourism, thus generating their desire to return to and recommend the destination. This therefore supports the argument in the paper that satisfaction in martial arts tourism has the ability to positively impact the tourism behavioral intention.

#### 5.1.2 Sports values can significantly and positively enhance tourism behavioral intentions

According to Yu et al. (2017), during the tourism process, the human mind, body and environment form a comprehensive, organic and closely interconnected whole. That is, tourists will actively integrate their perceptions, thereby giving meaning to various situations and thereby influencing their intentions in social behaviors. Thus, based on this, this study extends this finding to propose the hypothesis that the values of sports can also have a positive and significant impact on tourism behavioral intentions. In the context of martial arts tourism, the sports values possessed by college students or formed as a result of them, generate positive perceptions, which lead to choice of tourism behavior and judgments. However, when explaining this phenomenon earlier, it was mostly at a macroscopic level. Therefore, this study further suggests that values play a role in consumers’ choice behaviors. Values are the driving forces of behavior of individuals. The alignment of martial arts tourism with the sport values of the college student tourist will enhance their positive evaluation of the tourism experience which will eventually translate into tourism behavior intentions.

#### 5.1.3 Travel satisfaction can significantly and positively enhance sports values

Travel satisfaction is not merely the culmination of an experience, but the starting point for reshaping values. Previous research indicates that when individuals make decisions, their preferences for relevant attributes shift to support emerging choices, enabling confident decision-making (Simon and Spiller, 2016). From this perspective, when tourists gain satisfaction from martial arts tourism, it will encourage them to strengthen their sports values in order to maintain a balanced mindset. Generally speaking, the formation of values hinges on three interconnected stages: “needs-benefits-practice” (Tang, 2008). As Marx and Engels noted in The German Ideology: “The needs of men are their nature, and the manner in which they seek to satisfy these needs.” This indicates that tourism satisfaction reflects the fulfillment of certain tourist, and this fulfillment reinforces related sports values. This also provides philosophical support.

### 5.2 The mediating role of sports values

A review of recent studies, a clear trend can be observed: most studies focus on the direct relationship between the satisfaction of tourism and the willingness to go again. It is like they would go again even if they are satisfied. However, the question remains to be answered: what happens in between? What is the process through which tourists move from feeling satisfied to deciding to revisit? The intervening psychological process has largely been neglected. From this perspective, the problems in the field of martial arts tourism become even clearer. The sports value, which plays the role of the bridge between the past and the future in the minds of the tourists, should logically be an important clue. However, if we refer to the existing research in the field, few people have examined it as a mediating variable. How does it connect the two ideas of “satisfaction” and “revisiting.” has not been explored in depth. That is why we tried to place the sports value in the middle of the relationship and examines their mediating role from the economic, social, aesthetic, physical, and moral points of view.

#### 5.2.1 Economic

The mediating effect of economic values is positive and significant, indicating that satisfaction in martial arts tourism enhances participants’ perception of economic value, aligning with their value for money or even value exceeding expectations values, thereby strengthening behavioral intention. This finding resonates with Yi and Li’s (2025) conclusion that “college students exhibit pronounced tourism preferences and prominent economic rationality in tourism consumption,” Zheng et al. (2021) posits that “the student tourism market can be segmented into high-income and low-to-middle-income households,” Wu and Lian (2011), using Henan Province universities as a case study, analyzed that “for college students as a purely consumer-oriented tourism group, the key factor restricting their outbound travel is funding, which also becomes the most significant factor influencing their tourism consumption behavior.” Therefore, when college students experience satisfaction exceeding their expectations in martial arts tourism, it reinforces their economic value judgment that this activity offers high cost-effectiveness, thereby exerting a significant positive influence on their tourism behavioral intention.

#### 5.2.2 Social

The positive mediating effect of social values highlights the social function of martial arts tourism. As the public faces increasing mental pressure from work, family, and other aspects, social interaction within sports tourism has gradually become a focal point of public attention. This demand relates to social interaction, alleviating stress as well as managing emotions by engaging in sports tourism by members of the society. It is largely done under leisure and entertainment with the sports tourism process being utilized to enhance social relations and augment the sense of involvement and experience in sports tourism (Xu and Cai, 2025). At the same time, with the development of the internet economy, there are gradual changes in the context of knowledge and focus on social interaction among the youth. Human beings have changed into passive receivers of information to today, being the creators and sharers of information, and social support which is received in platforms is now a crucial source of information. Earlier, researchers have looked at t the ways in which online environments affect tourist behavior r such as the impacts of video social media participants (Han and Ming, 2021), social network communications (Song et al., 2019), recorded short video Douyin (Han and Ming, 2021), and the online reputation of a travel spot (Guo and Huang, 2021) on travel behavioral intentions. In online interactive settings, there is the development of stronger interpersonal relationships between the travelers and this leads to increased readiness to share and embrace information and thus shapes travel behavioral intentions. It is in line with the fact that (Mao, 2023) in this view on the influence of social returns on the process of travel behavior, the attitude of individuals and their subjective norms of behavior are not the main determinants of specific behavior, but more likely the views on the social returns on the behavior. As an example, social values derived during travel, e.g., social recognition, identity symbols, or relationship reinforcement, may shape behavioral decisions more strongly than conventional cognitive considerations.

#### 5.2.3 Aesthetic

Among all dimensions, the mediating effect of aesthetic value is the most significant and exerts a positive influence. This indicates that as a form of cultural experience, martial arts tourism possesses rich cultural connotations and strong competitive advantages. Wu and Shen (2018) suggest that compelling images, emotionally resonant text, and their layout, among other elements, possess the potential to evoke tourists’ aesthetic experiences. It is precisely this aesthetic experience that actively contributes to the formation of emotional engagement within tourism destinations (Guo, 2020). This finding is closely associated with the inherent aesthetic qualities of martial arts. Its elements such as rhythmic movements, cultural attire, body art and spiritual symbols can have a direct and profound impact on college students. This also aligns with Breiby and Slåtten (2015) proposition that “certain aesthetic qualities can have both direct and indirect effects on travelers’ intention to recommend a route and to visit similar routes.” Aesthetic value directly stimulates participants’ emotional responses. Aesthetic value is not merely a driving force for people’s emotional expression; it is more like a bridge that converts tourists’ satisfaction with their travel into behavioral intentions.

At the same time, martial arts are not merely a form of exercise but also an aesthetic process of self-shaping. The strongest mediating effect of aesthetic values in martial arts tourism stems from its precise alignment with the needs of contemporary university students—namely, achieving physical self-shaping through fitness activities like martial arts. Previous research indicates that physical exercise significantly influences individuals’ body self-esteem and aesthetic value perception (Wang H., 2024). Amidst the nationwide fitness movement, an increasing number of young people are enthusiastic about activities that improve physique and strengthen the body. Unlike common fitness equipment, martial arts routines consist of numerous individual movements characterized by complex directions, trajectories, rhythms, and body postures (Zhao et al., 2021). Members do not just become strong and develop their bodies, but also more flexible and coordinated. Therefore, when college students undergo the slight physical changes in the context of martial arts tourism, they are able to find the aesthetic gratification in self-enhancement. More importantly, martial arts aesthetic value goes beyond the usual emotional experiences, and it plays a strong role in the aesthetic education and the building of national self-confidence. On the one hand, being the representative of the best traditional culture in China, Chinese martial arts has its purposes in the cultivation of inner and external features and acquisition of the bottom by means of the higher. It focuses on cultivating feelings and adjusting personality, growing roots and shaping souls, and achieving a balance between hardness and softness and bringing form and soul together. Not only does it become an essential tool in the propagation of the spirit of the Chinese aesthetic education, but it also is a cultural boat filled with the spirit of aesthetic education (Dong et al., 2024).

Conversely, martial arts also inherited and contributed greatly to the cultural development of the entire nation due to the cultural nature. Martial arts routines are an inseparable part of traditional folk sports of china and are used as international symbols of Chinese culture serving as a hallmark and symbol of the nation. The aesthetic interpretation of martial arts has changed as follows in the words of the General Administration of Sport of China: martial arts is a traditional sport, grounded in the Chinese cultural theory; its core elements are offensive and defensive and relates to combat techniques and aims on the form, sparring and training (Hu, 2025). In that way, it was judged that the aesthetic perception of martial arts has deep roots in the traditional Chinese culture. The ultimate admiration and recognition of martial arts is, in essence, admiration and an appreciation of the ancient Chinese culture. This enhances the aesthetic values elicited by the martial arts tourism beyond the emotive experience into spiritual activity that instills national confidence.

#### 5.2.4 Physical

One discovery that cannot be ignored is that physical factors have had a negative impact in this process, and the effect is particularly significant. Intuitively, physical condition seems to have a positive impact on travel behavior. However, the reality is quite the opposite. Although travel satisfaction can enhance this factor of the body, the physical condition itself cannot stimulate people to have a stronger motivation for traveling. If martial arts tourism is merely regarded as a form of exercise, its true charm is likely to be overlooked. In fact, it is more like an immersive experience that requires complete dedication of both body and mind. Although improving physical fitness remains the reason why many people participate in it, as people gradually realize that this experience is not easy, the novelty and motivation will gradually fade away. Instead, it offers an immersive experience demanding wholehearted dedication. According to the Resource Conservation Theory (Hobfoll, 1989), people always tend to protect the resources they have painstakingly accumulated, such as energy and physical strength. Once martial arts tourism is regarded as a strenuous physical activity, participants tend to have concerns. They are worried that it might consume too much energy or disrupt their original plans. This is especially true for college students who have heavy academic loads and fragmented time. For them, once a leisure activity requires a lot of effort, it becomes an additional burden. The uniqueness of martial arts tourism lies in the fact that its physical challenges are unavoidable. This aspect is particularly problematic for those college students who already have reservations, as it can easily become a psychological burden and gradually turn into a resistance to martial arts tourism. Students tend to unconsciously magnify the difficulties and underestimate their own abilities. Before even starting, they lose the willingness to actively solve problems, ultimately resulting in behavioral rejection and psychological aversion (Yang, 2024). Furthermore, although the benefits of exercise are widely recognized, physical activity levels have generally declined across all age groups. This decline is particularly pronounced among adolescents and young adults (Moylan et al., 2022). And more and more people are inclined to choose convenient and comfortable activities. Therefore, the requirements of martial arts training for the body are different from those of daily activities of the participants. This difference in values regarding the body, in some cases, may even lead to some negative value associations.

#### 5.2.5 Moral

This line of argument can be supported by previous research. Related studies suggest that the genuine internalization of cultural meanings or values usually requires stronger situational immersion, real-world contact, and sustained reflection. For example, study-abroad research has shown that transformative learning is more likely to occur when overseas experiences are combined with reflective processing, whereas short-term exposure alone is often insufficient to produce deeper changes in perspectives or values (Behnke et al., 2014). At the same time, heritage tourism research has noted that some tourists seek genuine cultural understanding, while others are more oriented toward experiential or relatively superficial forms of engagement, indicating that short-term travel does not necessarily lead to deep cultural or ethical identification (McIntosh and Prentice, 1999). The other sources also indicate that heritage tourism is associated with a fairly high amount of psychological distance, and the enhancement of authenticity and engagement are required to suppress the distance and facilitate more meaningful-making (Scarpi and Raggiotto, 2023). The other potential reason is the way moral value was operationalized in the survey. The measurement items can be better tailored to the traditional morality connotations of martial arts in traditional settings of formal learning or long-term practice than in the short-term tourism settings. Consequently, the moral dimension may not have been adequately captured in responses of the participants. Future research may further refine the measurement of moral value in martial arts tourism, and comparing short-term tourists with long-term martial arts participants to determine the workings of moral value in various participation settings.

### 5.3 Implications of the study

Theoretical implications of this study are reflected in two aspects. This study first introduces sport values to the analytical framework between satisfaction in martial arts tourism and behavioral intentions that satisfaction has a direct effect in determining behavioral intentions as well as indirectly through mediating effect by sport values. This extends the explanatory line of behavioral studies in martial arts tourism. Second, the present study also demonstrates the multidimensional diversity in sport values. The results indicate that economic, social and aesthetic values have positive mediating roles with aesthetic value having the strongest effect; physical value having the suppressing effect whereas moral value has an insignificant mediating role. These results can suggest that various measures of sport values may not operate similarly in martial arts tourism and it adds to the comprehension of its psychological process.

The practical implications of this study are mainly reflected in the design of martial arts tourism products and the development of the college student market. First, martial arts tourism programs should not only improve overall satisfaction, but also pay greater attention to the cultivation of economic, social, and aesthetic value experiences in order to enhance college students’ willingness to revisit and recommend. Second, given that aesthetic value shows the strongest effect, related programs should place greater emphasis on the visual appeal, experiential quality, and cultural expression of martial arts so as to strengthen emotional resonance among young participants. Third, the suppressing effect of physical value suggests that program design should avoid excessive intensity and burden, and instead place more emphasis on appropriateness, enjoyment, and participation. Fourth, the non-significant role of moral value indicates that traditional martial ethics are not easily perceived in short-term tourism contexts; therefore, their communication may be strengthened through interpretation, interaction, and situational experience design.

## 6 Conclusion

Based on a questionnaire survey conducted among college students from five different provinces, namely Shandong, Shanxi, Anhui, Beijing, and Guangxi, this paper takes the 314 valid questionnaires finally collected as data support. After systematic organization and analysis, it ultimately reaches research conclusions with practical basis and theoretical support.

One of the most important findings of this study is that it has come up after a number of analyses with significant positive correlation between the satisfaction of martial arts tourists and their of course the following behaviors. It is slightly pertinent to mention that the effects of martial arts tourism are not situated in direct experiences that are elicited by tourism events as such, but also in the emotional arousal and spiritual arousal that can be induced by the tourism events o on inner perceptions and behavioral motivation of tourism. Such an internal satisfaction of participation can further enhance the desire that one has to continue the activity endlessly and to refer to others to do so too.

According to the results of the research, there is a deeper explanation for this phenomenon. In particular, five fundamental dimensions of sport values are critical in a mediating role. The positive impact of economic ones is that it is consistent with the pragmatism values of college students, and the positive influence of social ones means that martial arts tourism is slowly turning into a youth-related socialization. Of all the factors influencing it, aesthetic value demonstrates the strongest positive effect that can suggest the fact that the beauty of martial arts is the actual appeal of martial arts. It can also be noted that physical value had a significant suppressing impact meaning that as martial arts tourism is perceived to demand much physical effort or impose a heavy physical burden, it can weaken the willingness of college students to participate further. Moreover, the mediating role of moral value was also not substantial, which means that the traditional ethical connotations of martial arts might be hard to perceive and internalize by tourists in the context of tourist short-term martial arts tourism. This research in general has assisted in the interpretation of the psychological pathway in which satisfaction is converted to behavioral intention and empirically supported to explain the behavior of college students regarding their involvement in martial arts tourism.

### 6.1 Limitations

This study has some limitations. First, the sample was relatively unbalanced with a high number of second-year students and females represented. This level of concentration can impact the generalizability of the results. Moreover, sport values might vary depending on grade levels. The freshmen can put more emphasis on novelty and cultural experience compared to the senior students which can be more concerned with practicality, time expense, and stress relievement because of the pressure of employment. Second, the sample scope was relatively limited with only seven universities in five provinces being covered that cannot be regarded as the representative of the population. In addition, the instruments of research were also quite limited. The research was based entirely on the use of questionnaire survey and the conclusions made might be lacking depth in some areas. Future research ought to further improve sample balance, increasing the reach of sampling, and analyzing subgroup differences in terms of grade and gender through a combination of other research techniques, including interviews and observations.

### 6.2 Future research recommendations

Based on the findings and shortcomings of this study, we would like to propose several issues that can be further investigated in the future: First, at the sample size level, if future research wants to make further progress, it can put more effort into the quantity and scope of the sample. It should not focus only on a certain group of people; instead, it should take into account individuals from different regions, different ages, and different social backgrounds. Secondly, in terms of methodology, it is suggested that subsequent research can try more methods, such as interviews and observations. Only in this way can deeper relationships be discovered and more accurate conclusions be reached.

## Data Availability

The raw data supporting the conclusions of this article will be made available by the authors, without undue reservation.
